# The influence of adolescents' nutrition knowledge and school food environment on adolescents' dietary behaviors in urban Ethiopia: A qualitative study

**DOI:** 10.1111/mcn.13527

**Published:** 2023-05-07

**Authors:** Abreham Iyassu, Arnaud Laillou, Kassahun Tilahun, Fitsum Workneh, Sinksar Mogues, Stanley Chitekwe, Kaleab Baye

**Affiliations:** ^1^ Research Center for Inclusive Development in Africa (RIDA) Addis Ababa Ethiopia; ^2^ Nutrition Section UNICEF Ethiopia Addis Ababa Ethiopia; ^3^ Department of Psychology, College of Social Science Debre Berhan University Debre Berhan Ethiopia; ^4^ Center for Food Science and Nutrition Addis Ababa University Addis Ababa Ethiopia

**Keywords:** adolescents, dietary behaviour, Ethiopia, food environment, school nutrition

## Abstract

Adolescence is a critical period of physical, cognitive, and social development that needs to be supported with healthy diets. Dietary behaviours of adolescents can be shaped by their nutrition‐literacy and their interaction with parents and peers as well as their school food environment. Therefore, the present study aimed to assess factors that influence dietary behaviours of adolescents in urban Ethiopia. Sex‐disaggregated, focused group discussions (*n* = 70) were conducted in 36 private and government schools (*n* = 12/city) among adolescents (*n* = 432) 15–19 years of age in Addis Ababa, Bahir Dar, and Dire Dawa. Photovoice was applied in a subgroup of participants (*n* = 216) to gain further insights into adolescents' perception of their school food environment. Key informant interviews were conducted among school principals (*n* = 36). Adolescents had a relatively good nutrition knowledge and recognised the importance of diverse diets, but misperceptions also existed. They identified fruit and vegetables as healthy foods, but their consumption was deterred by food safety concerns. The adolescents identified foods high in salt, fat, and sugar, including processed/packaged foods as unhealthy, but still consumed them frequently due to their taste, affordability, availability and accessibility in and around schools. Both undernutrition and overweight/obesity were linked to social exclusion and bullying in school. Effective behavioural change communication is required to address common misperceptions. School nutrition programs should integrate water, sanitation and hygiene programs to ensure food safety. Regulations promoting healthy eating while discouraging unhealthy dietary behaviours are vital. Interventions to make nutrient‐dense and healthy foods available, accessible, and affordable are urgently needed to improve the nutrition and health outcome of adolescents.

## Key messages

- Adolescents had a relatively good nutrition knowledge, but misperceptions also existed
- Foods high in fat, sugar, and salt, but also processed/packaged foods were considered unhealthy, but were frequently consumed
- Unhealthy foods were widely available/accessible in the school food environment
- Comprehensive food safety and nutrition interventions are needed at schools
- Regulations for availability and advertising of unhealthy foods in and around schools are needed.

## Graphical abstract

Adolescents had a relatively good nutrition knowledge, but misperceptions also existed. —Unhealthy foods were widely available/accessible in the school food environment—Comprehensive food safety and nutrition interventions are needed at schools.

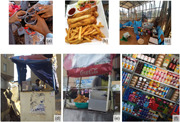

## 1 INTRODUCTION

An estimated 1.8 billion adolescents (ages 10–19 years) live in the world, 90% of which are residing in low‐ and middle‐income countries (Christian & Smith, 2018). In Sub‐Saharan Africa, adolescents account for close to one fourth of the total population (UNICEF, 2019). Adolescence is a critical period of physical, cognitive, and social development that requires adequate nutrition (Das et al., 2018). It is also marked by rapid growth reflected by a height gain equivalent to 15%–20% of their adult height and about 45% of their skeletal mass (Carel, 2004). Adolescents face a number of health challenges but because they are considered to be at a relatively healthy life‐stage, they have not been given adequate attention in global health and nutrition goals until recently (Patton et al., 2016).

The values, preferences and attitudes which are formed during adolescence, may shape lifelong dietary practices (Topolewska‐Siedzik & Cieciuch, 2018). Besides, while adequate energy and nutrient intake is necessary to support the rapid growth, healthy dietary habits are critical to prevent multiple forms of malnutrition (Das et al., 2017). Malnutrition during adolescence manifests in three broad groups of conditions: undernutrition, micronutrient inadequacies (deficiencies/excess), and overweight or obesity (WHO 2017).

Diets of adolescents in low‐ and middle‐income countries are characterised by a predominantly cereal‐based diets and limited consumption of animal source foods, fruits and vegetables (Keats et al., 2018). Numerous factors influence the dietary behaviours of adolescents, including brain development and understanding of matters that might affect health, the broader familial, socio‐cultural, and economic environment in which an adolescent lives, eats, studies, works and plays (Fox & Timmer, 2020). Adolescents are also influenced by factors in the food environment, such as aggressive advertisement of unhealthy foods influencing their dietary choices (Dillman Carpentier et al., 2020).

Although schools are recognised as a potential platform to improve diets of adolescents, little is known on how to best implement such interventions in urban settings in low‐ and middle‐income countries (Canavan & Fawzi, 2019; Downs & Demmler, 2020). School food environments which include food availability, prices, safety and advertising inside and around the schools can influence dietary behaviours (Gonçalves et al., 2021). In addition, factors related to individual's knowledge, perception, peers and families can influence what adolescents eat (Neufeld, 2022).

Ethiopia has a high burden of undernutrition, and in recent years a surge in overweight and obesity has been observed, particularly in urban areas (Baye & Hirvonen, 2020). In 2016, 21% of women (15–49 years of age) in urban areas were overweight or obese (Baye & Hirvonen, 2020). The meta‐analyses by Gebrie et al. (2018) estimated a relatively high prevalence (11.30% [95% confidence interval: 8.71%, 13.88%]) of overweight and obesity among children and adolescents, while at the same time undernutrition remains a significant problem (Baye & Hirvonen, 2020). These figures suggest the need for interventions that can address both forms of malnutrition through the promotion of healthy diets that is a shared driver for both forms of malnutrition (Pradeilles et al., 2019).

In Ethiopia, the few studies investigating factors influencing dietary behaviours of adolescents were either conducted in rural areas or the capital, Addis Ababa (Handiso et al., 2021; Trübswasser, Baye et al., 2021). Other studies have focused on the urban home food environment of adolescents. However, more detailed investigations of the school food environment and dietary behaviours of adolescents from multiple cities, where diets are changing due to transforming food environments is critical. Such information is essential for the design of effective urban school nutrition interventions (Pineda et al., 2021).

Therefore, the present study aimed to assess factors that influence dietary behaviours of adolescents in private and public school students in three Ethiopian cities: Addis Ababa, Bahir Dar, and Dire Dawa. We also aimed to assess the interaction of adolescents with their school food environments to inform future interventions that aim to improve adolescents' diets. The conceptual framework of food systems for children and adolescents served as a basis for our study (Raza, 2020). This framework was applied and found useful to assess adolescents' food environment in Ethiopia (Trübswasser, Baye et al., 2021). Our study focused on the two food environment domains: external and personal. The external food environments in our study included food availability, advertising, prices and safety at school canteens and vendors outside the school. The personal food environment elements referred to affordability, convenience, and access. Our study also assessed behaviours of the adolescents, which also included their knowledge, practices, and desires.

## 2 METHODS

### 2.1 Study area and population

The study was conducted in three cities of Ethiopia, the capital Addis Ababa and two secondary cities: Bahir Dar, and Dire Dawa. According to estimates from the worldometers Addis Ababa (~3,000,000) and Dire Dawa (~250,000) are the top two most populated cities in Ethiopia (Worldometer, 2021). Bahir Dar is the 5th largest city in Ethiopia, with an estimated population of 170,000 residents. In each city, six private and six public secondary schools were selected, informed by the respective education bureaus of the cities.

### 2.2 Sampling

The study population consisted of adolescents between 15 and 19 years of age. Study participants attended grades 9–12 in private and public secondary schools of Addis Ababa, Dire Dawa, or Bahir Dar cities. While selecting private and public schools in each city, a purposive sampling technique was employed with the aim of representing various socioeconomic background. The principals helped with the selection of participants as they had good information on the socio‐economic status of the children's family. This approach was also a practical way to get good representation without singling out students based on a screening question as existing documentation was not available.

Schools were selected from both the centre and the peripheral areas of cities. Inner and outer cities often represent varying food environment and socio‐economic status, although there is little documentation on this but is rather a lived reality. For Addis Ababa, inner cities refer to built‐up areas existing pre‐2003; whereas outer cities are urban expansion areas that have developed in the last two decade (post‐2003), as documented in Moisa & Gemeda (2021).

Purposive sampling was employed to select students (6 girls and 6 boys) in each school. After briefing school principals about the objectives of the study, they assisted in the selection of students with different socio‐economic backgrounds across all grade levels and ages groups between 15 and 19 years. Key informant interviews (KIIs) were also conducted with school principals or their deputies on their perception of adolescents' dietary behaviours, the school food environment, as well as on relevant school regulations and policies. In total, this study was conducted in 36 (18 private and 18 public) secondary schools in Addis Ababa, Bahir Dar, and Dire Dawa. A total of 432 adolescents and 36 school principals participated in the study.

### 2.3 Data collection

Students' characteristics related to school type, age, grade level, and means of transportation to school were captured by the focused group discussion (FGD) facilitator. School principals were asked about the number of students, age, and sex distribution of students, number of students per class, total number of teachers, and the presence of parent‐teacher association in the school. All interviews and FGDs were conducted face to face between April to June 2021. Before starting the discussion, a round of introduction was conducted, which helped capture information on the individual participants.

All interviews and discussions were audio‐recorded and conducted by six interviewers, who are experienced qualitative researchers, who underwent a 2‐day training on the objectives of the study and the data collection methodologies. The training included sessions on how to obtain consent/assent, taking pictures after obtaining permission, and keeping confidentiality of the information obtained. The team had a background in psychology, public health, social work, and nutrition, which led to minimise potential biases related to their positionality. The researchers are familiar of the context, but were not from the community giving them a more distant and objective view. The discussion guide was designed in such a way that daily debriefings with supervisors were conducted to address issues as they arise, in a harmonised way The three independent researchers discussed and compared codes and examined discrepancies in coding to reach consensus and establish a standardised method.

#### 2.3.1 FGDs and KIIs

A semi‐structured FGD guide was applied to gather information from students about their nutrition knowledge, their perceptions about healthy diet, food consumption patterns, and factors in the school feeding environments influencing students' dietary behaviours, which also included food safety and hygiene. Each FGD was conducted with 6–8 participants with girls and boys in separate groups. At every school two FGDs were conducted; one with female and the other with male participants. The FGD used a guide including questions to assess nutrition knowledge related to healthy and unhealthy diets and how they related to different types of malnutrition. The FGD participants were also asked about the available food at the schools, use of their pocket money for food in and around the schools, and potential barriers and enablers to the adoption of a healthy diet.

The FGD was complemented with KIIs with 36 (one per school) principals or deputy principals of all schools. The interviews included questions about the principal's perception of adolescents' dietary behaviours, the school food and nutrition policies and barriers and enablers to healthy diets within the school compound. Principals were also asked about food safety and hygiene issues within the school.

#### 2.3.2 Photovoice

To triangulate the evidence from the individual interviews, the study employed a participatory action research method called Photovoice to assess adolescents' dietary practices and perceptions of their individual, social influences, external and personal food environment at school and home. Photovoice is a methodology, in which participants take photographs in response to a research question. The photographs are then analyzed by selecting the most relevant photographs for the topic; contextualising and explaining the context of the photographs; and codifying/identifying the issues, themes revealed in the photographs (Wang & Burris, 1997).

After participating in the FGD, students were identified for the Photovoice sub‐study. In each site, pairs of adolescents (*n* = 6 per school), were selected to be part of the photovoice discussion. The selection of the students was supported by the principal based on expressed interest to take part in the study, but also considering students': access to a smart phone and consent to take pictures during out‐off school periods. Students received basic instructions in the Photovoice method, ethical issues related to taking photographs and were tasked to take a maximum of 10 photographs reflecting what influences their dietary behaviors in the school food environment.

Students were asked to take photographs answering two specific questions: (1) What in the school (internal and external) environment helps you to eat healthy? (2) What in the school (internal and external) environment prevents you from eating healthy?

After taking pictures, the participants had to select two photographs that best answered the questions with the support of the facilitators. The selected photos were then used for photo‐elicit interviews. The interviews were always conducted with two participants, were held in the local languages, and guided by a semi‐structured tool, using questions, abbreviated as “SHOWeD” (1) What do you See here?; (2) What is really Happening here?; (3) How does this relate to Our lives? (4) Why does this problem or this strength exist? (5) Why is the situation like this? and (6) What can we Do about this? (Wang et al., 1998).

**Figure 1:**
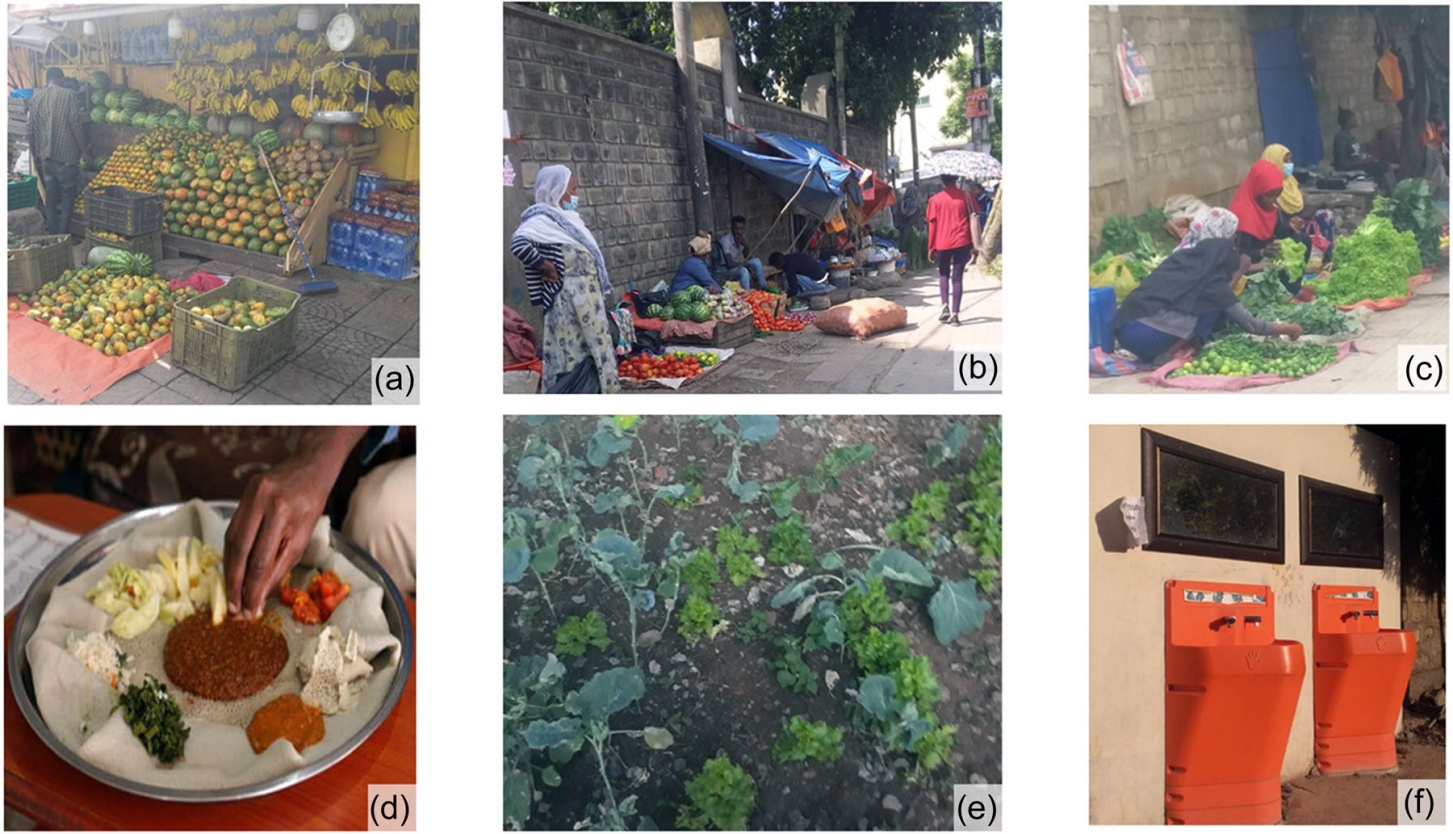
Example of pictures from the photo‐voice illustrating enablers for healthy eating. (a–c), Fruit and vegetable available around the school (Addis Ababa‐Shola market area); (d) an example of a traditional diverse diet considered healthy; (e) school gardening; (f) hand‐washing post with soap dispenser in Addis Ababa.

**Figure 2:**
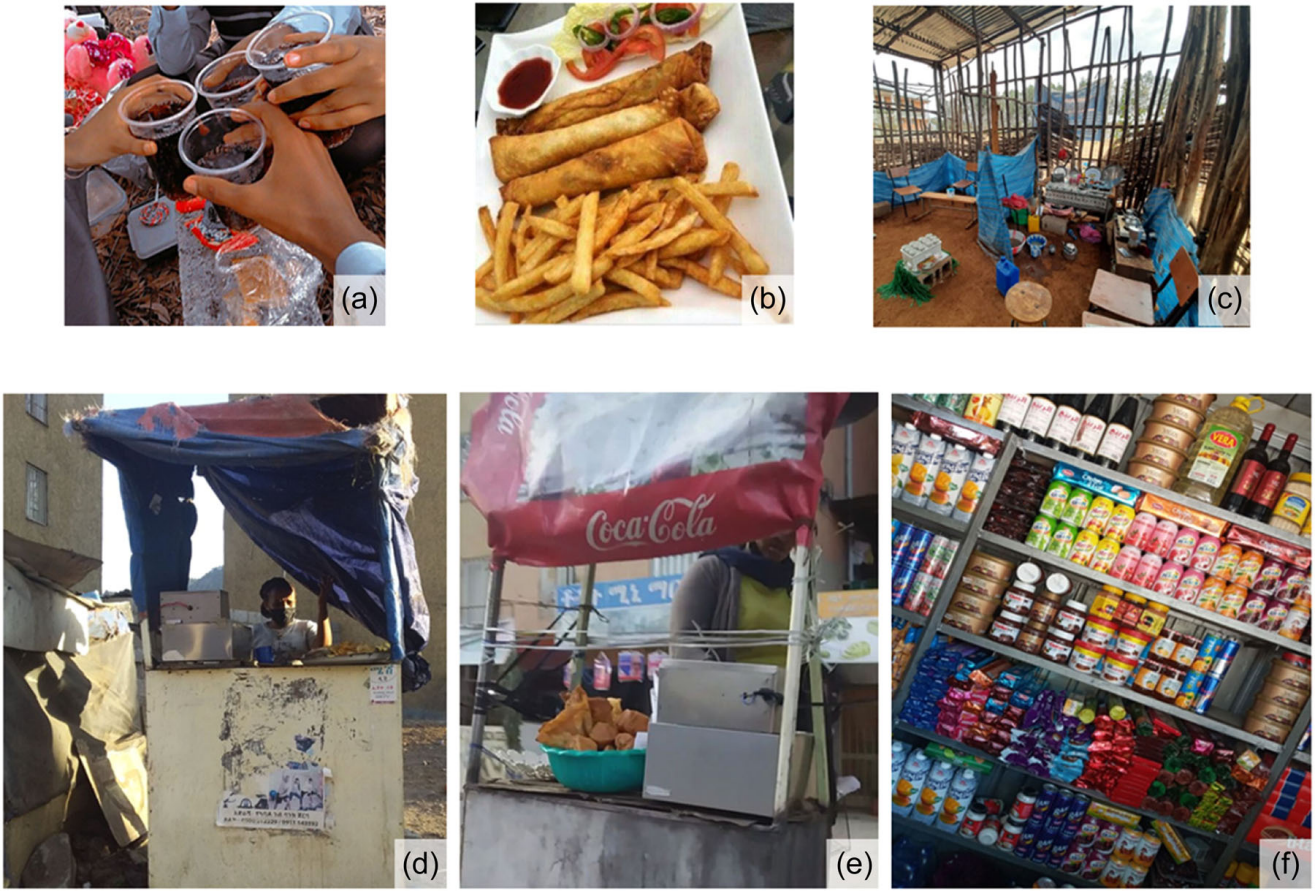
Example of pictures from the photo‐voice illustrating barriers for healthy eating. (a) Sharing of sugar‐sweetened beverages in a school in Addis Ababa; (b) Fried foods available around the schools in Addis Ababa; (c) cafeteria in a government school in Bahir Dar; (d, e) unhygienic fried street foods around schools; (f) kiosk selling almost entirely processed foods around a school in Dire Dawa.

Discussions were recorded, transcribed, and analyzed. Examples of illustrative pictures are presented in Figures 1 and 2. The audio‐recordings were transcribed verbatim and then translated to English. Accuracy of the translation was checked by back‐translation from English to Amharic.

### 2.4 Data analyses

Transcripts of the interviews and the FGD from all the sites were analyzed independently by three researchers. The researchers discussed and compared their coding and examined discrepancies to reach consensus and establish a standardised coding approach. The data were arranged by school, study site, and sex and compared against each other to examine similarities and differences. The transcripts were coded using a qualitative data analysis software, MAXQDA (Kuckartz & Rädiker, 2019). A priori codes based on the *Innocenti* framework were used to code the transcripts from the FDG and the photovoice discussions (Table 1). In addition to concepts from the Innocenti framework, we added codes related to social factors such as peer or parents' influence that we identified from the literature as relevant to adolescents (Neufeld, 2022). Additional codes that emerged from the data were included in the coding framework as part of the coding process (Saldana, 2009). Data were then categorised by relevant themes and subthemes (Ryan & Bernard, 2003).

**Table 1:** Main themes and subthemes guiding and emerging from the analyses.

A priori themes	Main themes adopted	Sub‐themes	Description
Behaviour of adolescents	Dietary behaviours and nutrition knowledge	○ Eating patterns ○ Taste and preference ○ Knowledge	Food procurement, preparation, supervision, and eating practices of adolescents. Eating behaviours are the consumption practices of adolescents. They reflect what and how they eat, and are influenced by adolescents' eating patterns, taste preferences, nutrition‐knowledge, appetite, level of physical activity, as well as psychosocial factors.
Social factors (or social env)?		○ Peer influence ○ Parents' influence	
Personal food environment	Personal and school food environment	○ Convenience ○ Source of nutrition information ○ Promotion and advertisement ○ Accessibility ○ Purchasing power (pocket money amount and use) ○ Inside school food environment ○ Outside school food environment ○ School food policy/guidelines/regulations ○ Food vendors ○ Accessibility, affordability and availability of foods in and around schools ○ Food safety	Individual and household level factors that consumers bring to the food environment, such as purchasing power, access, convenience and desirability, and inform why people choose to procure the foods that they do.
School food environment			External food environment includes the retail and commercial markets, schools, and informal vendors, among others, where consumers interface with food. It reflects aspects related to availability, food price, marketing and advertisements, and vendor and product properties (e.g., vendor hours, food offered, etc.).

### 2.5 Ethical considerations

Ethical clearance was obtained from the College of Natural and Computational Sciences, Addis Ababa University. Support letters were obtained from the respective education bureaus, and permissions were obtained from each school principals. After explaining the purpose of the study, school principals had to give consent to participate in the KII. Written consent was obtained from the participants' primary caregivers communicated through the school principals. Assent was obtained from the participants by the researchers before the interviews.

## 3 RESULTS

### 3.1 Dietary behaviour and nutrition knowledge

Most adolescents referred to good nutrition as a balanced diet that allowed them to meet their nutrient requirements. Notions of diet diversity, amount and timing of food consumption were also mentioned.

“Nutrition refers to using different types of food with varying nutrient contents based on the need of our body.”

Adolescent girl, private school, Addis Ababa

“…A balanced and a healthy diet is when a person consumes food that includes vitamins, carbohydrates, protein, fat and minerals…”

Adolescent boy, private school, Addis Ababa

“Diversifying foods is helpful since we get different nutrient from different foods and that is good for our health and physical appearances.”

Adolescent girl, public school, Dire Dawa

“Good nutrition consists of dairy and meat products, and different fruits like papaya, banana,…”

Adolescent boy, public school, Dire Dawa

Generally, adolescent girls in our study had a more comprehensive understanding of healthy diet than boys as they were able to mention multiple dimensions like safety, quantity and quality. Adolescent girls also mentioned the importance of food safety, hygiene, and the need to eat more “natural” and “organic” foods.

Both the FGDs and the KIIs revealed that fruit and vegetables were considered as key components of a healthy diet; hence, participants recommended consuming such foods on a regular basis as snacks or main meals. Participants suggested that efforts should be made to eat from different types of fruits and vegetables.

“People who frequently incorporate fruit and vegetable as part of their diet are more likely to be healthy and live longer. We can get proteins, vitamins and carbohydrates from consuming fruit and vegetables… Having fruits and vegetables can help us build our immune system and support us fight diseases. They also support the digestive system if taken as desserts. So I think it is very useful to have fruits and vegetables more often…”

Adolescent boy, Private school, Addis Ababa

“…No doubt that they [fruits and vegetables] are healthy. But if we eat them frequently, they have their own side effects. We must take them in the right proportion. We should have varieties of such foods. Eating the same fruit daily might have a negative effect on us…”

This is further explained by the following example:

“…consuming avocado regularly can lead to overweight, whereas salad and cucumber help us lose weight. Thus, we need to be selective while taking fruits and vegetables daily.”

Adolescent girl, Private school, Addis Ababa

Overall, the students in all the three sites and irrespective of the type of school, had a relatively good knowledge about good nutrition, healthy diet, and their consequences. The main misperceptions are presented in Table 1. Misperceptions related to eating differently depending on an individual's blood type were widespread and could also be a reason for consuming fewer fruit and vegetables.

“It is healthy to have fruits and vegetables. But, I would suggest people to check their blood type and have their dietary choices based on what is compatible to their blood type. My blood type is A…and I know avocado is good for me. People who have a blood type of “O” are not advised to eat Avocado. So people should make their choices based on their blood type. It would be meaningless to consume fruits and vegetables that don't match with our blood type…”

Adolescent boy, Public school, Addis Ababa

Most adolescent boys considered to have outgrown the period when they need to bring their lunch to school. Even if some students still want to bring their lunch, the peer pressure to purchase food is high which kept them from brining food to school.

“Friends are influential. If you bring lunch and your friend doesn't, he might tell you to stop bringing lunch and buy something here. Then you will start buying food.”

Adolescent boy, public school, Bahir Dar

Foods that are fried, high in fat, oil, sugar, salt, and processed/packaged foods were identified as unhealthy foods. However, some students argued that there is no bad or unhealthy food, and that the amount and frequency of consumption was more important.

“… during the holidays, we use too much fat and oil in our foods, we can even see the changes in our body fat…”

“I do not think that there is such a thing as a unhealthy diet; otherwise, we would have not survived…For example, injera [Ethiopian staple food] is only rich in carbohydrates, and thus could have been considered as unhealthy food… However, even if it is poor in protein and other nutrients, we have been consuming this food for many years without any problem…so instead of referring such foods as unhealthy, they should be identified as nutrient poor instead…

Adolescent boy, Public school, Addis Ababa

When asked about adolescents' perceptions on available processed/packaged foods in the school food environment, adolescents considered those foods as generally unhealthy. Adolescents were concerned about the additives in packaged foods which are used to preserve the food, enhance its flavour and colour. The adolescents were most concerned about the nature (natural vs. synthetic) of the ingredients. Manufactured products were also considered, by most adolescents, as products that lost their “organic” and “natural” flavour.

Adolescents believed that the high amount of added sugar and salt in packed/manufactured foods or drinks lead to loss of appetite, tooth decay, or diabetes. The adolescents also believed that consumption of chocolate was a major cause for overweight, whereas sugar‐sweetened beverages (SSBs) like *Coca cola* and *Pepsi* were associated with loss of bone strength. In contrast, *Mirinda*, another brand of SSB, was perceived as beneficial for patients who feel weak, are anemic or those who donated blood. Therefore, *Mirinda* was considered good for anemia and for those that are underweight, but to be avoided by those who are overweight or diabetic.

The most frequently consumed packed or fried foods reported by adolescents were biscuits, chocolate, chips/french fries, and instant noodles; whereas, frequently consumed packed drinks included fruit juices, sodas, milks, and bottled water. Despite the wide recognition that some of these packed foods are unhealthy, a large proportion of adolescents were still consuming them because they were cheaper and tasty to them.

“I don't advice anyone to consume packed foods. Major problems are seen in relation to the chemicals added, the poor ways of transporting and storing them. Personally, I do not allow my children to consume packed foods. But in the school, we see a lot of students having packed foods and drinks. The cafeteria sells packed biscuits and we see a lot of students having them during the break and lunch time. When I asked them why they used to buy biscuits, they say it is because they are cheap”

Principal, Public school, Addis Ababa

“…though we have the awareness about the harm of these foods, we are still consuming them.”

“…the problem is not awareness, but lack of dedication and commitment to practice what we know.”

“They [packaged commercial foods] are not healthy because: one, we are not sure if they were contaminated during production; two, people in our society are not conscious about expiry dates; and three, the transportation facilities and stores do not fit standards, especially for packed fruit juices and drinks.”

Adolescent boy, private school, Bahir Dar

Some argued that packaged foods had the advantage of containing essential nutrients, and they also considered the high energy content as important for adolescents that are physically active. This was in relation to fortified packed foods that contain essential nutrients.

The school principals raised concerns over adolescent girls skipping meals and avoiding eating because they were overly conscious of gaining weight, which could lead to unhealthy dietary behaviours.

“… There are some female students in the secondary school that are too conscious of their looks and who may be afraid of having food. They may opt to skip their dinner because they are afraid of gaining weight. But that leads to weight‐loss and under nutrition. If we measure their BMI, we will definitely see students that are under weight.”

School principal, private school, Addis Ababa

Unhealthy foods, such as high salt‐, high fat‐ and high sugar‐containing foods were identified to be linked to poor immunity, underweight, or overweight, all of which were described as having negative health impacts. While underweight was associated with poor immunity and resistance to disease to the adolescents, overweight was related to diseases such as cardiovascular diseases, diabetes, and stroke.

“Overweight can bring gastritis, hypertension, and other diseases. Overweight is an illness by itself.” … “Overweight….has consequences like heart problems, diabetes, hypertension, excessive cholesterol, and mental problems.”

School principal, public school, Bahir Dar

Students from Addis Ababa and Dire Dawa also referred to some students as being “addicted” to *Coca Cola*. However, consumption of sugar‐sweetened beverages appeared more dominant among students from Dire Dawa and Addis Ababa, and those from higher economic status (i.e., private schools). In Dire Dawa, consumption of sugary foods considered traditional, like *Baklaba, Mushebek*, and *Halewa*, were reported as being consumed frequently. It is also common to have dates and cookies during the Ramadan fasting season.

### 3.2 Food environment

The school principals had divided opinions regarding the primary cause of overweight/obesity. Some believed that the primary cause of overweight/obesity was the lack of physical activity, others considered unhealthy eating as the primary cause. This can have implications into the action they prioritise to improve the school food environment. Differences were also observed by location, where in Bahir Dar and Dire Dawa, school principals believed that physical inactivity was the root cause; whereas, in Addis Ababa, unhealthy eating was mentioned as a primary cause. Low physical activity among adolescents was also believed to have been exacerbated by the COVID‐19 pandemic, as illustrated by the quotation below:

“…in economically better‐off families, children are restricted to move anywhere beyond school and home. With the COVID‐19 pandemic, the lack of physical exercise is leading to overweight.”

School principal, private school, Dire Dawa

Adolescents were also concerned of the social stigma associated with being underweight/overweight. They reported that being underweight or overweight excluded them from being part of sport teams as they are considered to be weaker than normal‐weight students.

**“…**When people are overweight or underweight, other people will ridicule and bully them and this again will cause boredom and depression…being overweight can lead people to feel insecure and lose self‐confidence …”

Adolescent boy from private school in Addis Ababa

The source of nutrition information mentioned by adolescents was primarily from biology classes from grade 7 to 12. In addition, physical education classes, (social) media, peers, health professionals were mentioned as sources of information.

“I learnt about nutrition when I was in grade nine…from biology and physical education textbooks… however, in the new curriculum, information on nutrition is included starting from grade six…”

Adolescent boys, public school, Addis Ababa.

“…our parents tell us not to consume foods high in fat… sometimes, we also hear about it from family members and people in our neighborhood when they talk about how some food items may be bad for our health. Older people also talk about having to avoid certain types of food because they have a disease and the doctors told them not to consume certain food items…”

Adolescent boys, private school, Addis Ababa

“Health professionals also advice sick people to eat fruit and vegetables, those foods are healthy…”

Adolescent girls, public school, Dire Dawa

Most students mentioned that they also rely on media, both conventional (TV/radio drama and advertisements) and social media, as a source of information. Girls in Dire Dawa mentioned that they watch videos from YouTube:

“I watch a movie called yehualachin Tarik [also known as yegna]… I learn about food from it. It passes messages about food. I watch it from YouTube and it teaches about diversifying food,”

Adolescent girl, public school, Dire Dawa

Although most schools have health clubs, they rarely focus on nutrition‐related issues. Instead the primary areas of focus were reported to be on reproductive health, HIV/AIDS, and prevention of drug and substance abuse. Similarly, the few schools that have health professionals (e.g. nurse) focus on providing first aid and supporting menstrual hygiene.

Most adolescent boys considered to have outgrown the period when they need to bring their lunch to school. Even if some students still want to bring their lunch, the peer pressure to purchase food is high, which kept them from bringing food to school.

“Friends are influential. If you bring lunch and your friend doesn't, he might tell you to stop bringing lunch and buy something here. Then you will start buying food.”

Adolescent boy, public school, Bahir Dar

Although most adolescents perceived packaged/processed foods as unhealthy, their wide availability in and around the school and their desirable taste made it difficult for them to resist.

“…though we have the awareness about the harm of these foods, we are still consuming them.”

“…the problem is not awareness, but lack of dedication and commitment to practice what we know.”

Adolescent boy, private school, Bahir Dar

The most frequently available and consumed packed or fried foods were biscuits, chocolate, chips/french fries; whereas, frequently consumed packed drinks included fruit juices, sodas, milks, and bottled water. Despite the wide recognition that some of these packed foods are unhealthy, a large proportion of adolescents were still consuming them because they were cheaper and were widely available in and around the schools.

Adolescents mentioned that as long as the expiration dates were checked and the packaged products were bought from “trustworthy” sources like supermarkets, they could be considered as healthy.

“Packed foods are produced to provide more benefit to us. That is why they are processed in factories and made available to us in wider markets. The chemicals added to them could also be beneficial to our health and I think it is useful to consume packed foods and drinks… it is up to us to check their expiration date and ingredients. If they are not expired, they have the required ingredients, and are stored in a safe environment, it wouldn't be a problem to consume these foods.”

Adolescent Boy, Public school, Addis Ababa

School principals' confirmed that students were highly attracted to sugary foods like doughnuts, cakes, and soft drinks, which were also sold in the schools' cafeteria.

French fries, *pasty*, *bonbolino* (i.e., beignet/fried dough), and *sambussa* were commonly available and accessible. Fried foods were considered as unhealthy, mainly because of the way and the hygiene conditions in which they were prepared rather than their nutrient content or composition.

“… to increase their [street‐vendors] profits, they use cheap, lower quality oil, and use the same oil repeatedly for two to three days. That is why I am saying fried foods are not healthy and shouldn't be consumed.”

Adolescent boy, Public school, Addis Ababa

Students were also worried about the hygiene of the fried foods, since once fried, they were stored uncovered until purchased; and therefore, exposed to environmental contaminants. Besides, the students complained that the food handlers did not maintain personal hygiene, nor did they work in a sanitary/hygienic setting. Consequently, the students were more concerned about the acute health effects like typhoid than the high content of oil or salt, as they considered themselves less at risk to chronic disease at their age.

Food safety and hygiene were also mentioned as important factors for fruit and vegetable consumption. Typhoid and cholera have been reported as diseases that could potentially be transmitted through contaminated fruits and vegetables. Adolescents also identified the use of pesticides to grow vegetables as a factor preventing them from consuming fruit and vegetables. This was reflected in the FGD by an adolescent boy in Bahir Dar, as follows:

“…There is a high risk for fruits and vegetables to be contaminated during transportation to markets and the way merchants store or sell them. Hence, there is an increasing health problem related to fruits and vegetables. Care must be taken. …We need also to take care while consuming fruits and vegetables grown with different pesticides. That said, it is healthy to consume fruits and vegetables that grow free of pesticides of any sort…”

Adolescent boy, Private school, Bahir Dar

Most schools did not have a dedicated space where students can sit and eat. Most had cafeterias, but the food options sold in the cafeteria were limited to processed/packaged or fried foods. The cafeteria's selection of these foods seemed to be in line with the preference and purchasing power of the adolescents. In addition, due to the limited space dedicated for students to eat their lunch, the poor hygienic conditions of the cafeteria and the intermittent water supply in the school compound, packaged foods were considered as a safer, cheaper and more convenient options. Besides, these foods were also more convenient for the adolescents to grab and eat anywhere.

“We prefer to buy doughnuts and chips because they are cheap. The cost of fruits and doughnut is incomparable, fruits are expensive and they are not affordable for students…besides, fruits are not easily available”

Adolescent boy, public school, Addis Ababa

The location of schools is also a critical factor influencing students' dietary behaviour. Many kiosks and cafeterias can be found in front of schools. These outlets primarily sell processed foods high in fat, sugar, or salt.

“Merchants look for a strategic place for their business. Like it is common to access drug stores around hospitals, it is also common to access stationary, kiosks and junk food outlets in school surroundings … In front of the shops, you can find a frying machine. It is difficult to escape these foods if you have money.”

Adolescent girl, public school, Addis Ababa

The KII with school principals revealed that although sporadic, the private sector supports them by sponsoring events (e.g., inter‐school sport competition). However, these were also used as an entry point to advertise their food products.

“I remember a burger house that sponsored an event in the school… students competed and those ranked in the top three were invited to have whatever they wanted from the burger house…this was also a promotion for the company,”

KII from private school in Dire Dawa

“…a soft drink company came to the school a few years ago to do advertising…”

KII from public school in Addis Ababa

Table S1 summarises examples of identified behaviours as well as personal and school food environment issues that need to be addressed by future interventions.

## 4 DISCUSSION

Our study aimed to assess factors that influence dietary behaviours in selected urban schools in Addis Ababa, Bahir Dar, and Dire Dawa. The dietary choice of adolescents is influenced by peer influence, nutrition knowledge and perceptions, availability/accessibility and safety of foods. Adolescents had a relatively good nutrition knowledge, recognising the importance of diverse diets to meet their nutrient requirements. They perceived fruit and vegetables as healthy; whereas food high in salt, fat, and sugar, including processed/packaged foods were perceived as unhealthy. Food safety and hygiene concerns were often considered more important than nutrient content of foods. The internal school environment was not conducive for sitting and eating a meal, and the foods available in and around the schools were generally considered as unhealthy. School canteens and vendors around schools mostly offered inexpensive processed or fried foods. Advertising and sponsoring food companies promoting unhealthy foods were also important factors. Affordability, availability, accessibility, peer pressure, and food safety were among the major factors influencing food choice.

Our study showed that adolescents had a relatively good nutrition literacy, despite the fact that the inclusion of nutrition information in the curriculum was limited. This is in line with findings from a recent systematic review of qualitative studies among adolescents in low‐ and middle‐income countries (Trübswasser, Verstraeten et al., 2021). Adolescents' accessed nutrition‐related information from various sources, which at times did not provide accurate information. The belief that blood types should be considered in dietary choices, or that *Mirinda* (a brand of soft‐drink) can prevent anemia were examples of misperceptions that need to be addressed by future behavioural change communications.

The relationship between food consumption and the risk of undernutrition and overweight was clearly understood by adolescents. Although the risk of non‐communicable diseases (NCDs) related to unhealthy eating was recognised, students were more concerned about the social exclusion and bullying associated with being underweight or overweight (Van Geel et al., 2014). Such victimisation can in turn fuel obesogenic behaviours by restricting engagement in social and physical activities (Puhl et al., 2020). There was also tendency to consider that the role of healthy diets can be replaced by a physically active lifestyle and vice‐versa. Indeed, such dichotomies between healthy diets and physical activity have been reported elsewhere (Trübswasser, Verstraeten et al., 2021), and such views could have served as an entry point for the SSB industry to shift the narrative related to the prevention of overweight, to focus away from diets towards physical activity (Greenhalgh, 2021). Consequently, by promoting and sponsoring sporting events, the SSB industry claims to have served its social responsibility of preventing overweight and obesity (Herrick, 2009). Similarly, we also found that the SSB industry sponsored major sporting events like the inter‐school soccer games, which was qualified by the school principals as advertising (Murphy et al., 2015). This calls for stricter regulations and implementation of legislations that restrict the promotion, advertising, and selling of unhealthy foods in and around school environments, as implemented in cities like Rio de Janeiro (Hawkes, 2004).

Packaged/processed foods were generally considered unhealthy, but were still frequently consumed as they were available, accessible, and affordable to adolescents. The lack of (indoor) sitting space to eat and the dusty physical space in some schools encouraged students to buy processed foods that they considered more convenient to consume “on the go”. In addition, considering the limited purchasing power of the adolescents and their taste preference towards high sugar and fried foods, most school cafeterias only served cheap processed/fried foods, making it difficult to adopt a healthier diet. Additional factors encouraging the consumption of unhealthy snack foods were the food safety concerns, exacerbated by the intermittent water supply, which could limit fruit and vegetable consumption (Trübswasser, Verstraeten et al., 2021). Social norms and peer pressure related to home‐prepared, packed lunch could also lead to adolescents consuming more packed foods from the school cafeteria or surrounding vendors.

Infections related to poor food hygiene were of more immediate concerns than the long‐term consequences (e.g., NCDs) of unhealthy eating (Trübswasser, Verstraeten et al., 2021). This finding is in line with findings from various African countries (Pradeilles et al., 2021), and call for an integration of food safety and nutrition interventions in schools, possibly through interventions targeting vendors in the school food environment. Healthier alternatives that address the demand for convenience and safe food could also be considered.

## 5 STRENGTHS AND LIMITATIONS

Our study, which included both private and government schools, different cities, equal numbers of boys and girls and a relatively large sample size, allowed us to capture misperceptions and factors that influence food choices among adolescents in urban schools in Ethiopia. This was further supported by applying the photovoice method that allowed to integrate the adolescents' views and perceptions related to their food environments. Although efforts were made to include schools representing different socio‐economic status and urban contexts, our findings cannot fully reflect the situation in these three cities. While the school food environment and the knowledge of adolescents was the focus of the study, it should be recognised that the home‐environment also plays a key role in shaping food choices of adolescents. Parental influence on adolescents can be very strong on their dietary behaviours (Neufeld, 2022), which our study was not able to fully assess. The selection of the photovoice participants, facilitated by the principals, might have also led to selection biases. Future studies should aim to understand how home‐ and school food environment influence dietary choices and behaviours.

## 6 CONCLUSION

The findings of our study highlighted the role of the personal and school (external) food environment on dietary choices of adolescents. The dietary choices of adolescents are influenced by social influences (peers), food perception, food safety, and the availability/accessibility of foods. Understanding these drivers is critical for the design of school nutrition interventions, which should address the various food perceptions that can be modified through effective behaviour change communication as well as regulatory policy actions. School nutrition interventions should also integrate nutrition and food safety. Regulations and interventions that make healthy foods more available, accessible, and affordable while restricting advertising of unhealthy foods and beverages in and around the school are direly needed. The design of such interventions can benefit from participatory approaches such as the photovoice that can help integrate the adolescents' views and perceptions.

## AUTHOR CONTRIBUTIONS

Abreham Iyassu, Kaleab Baye, and Sinksar Mogues conceived the study. Abreham Iyassu, Kassahun Tilahun, and Fitsum Workneh executed the field work; Abreham Iyassu, Arnaud Laillou, Kassahun Tilahun, and Kaleab Baye wrote the paper with inputs from Stanley Chitekwe and Sinksar Mogues. All authors read and approved the final manuscript.

## CONFLICT OF INTEREST STATEMENT

The authors declare no conflicts of interest.

## Supporting information

Supporting information.

## Data Availability

this is a qualitative survey and thus not applicable.
