# Supplementary material for: The influence of adolescents' nutrition knowledge and school food environment on adolescents' dietary behaviors in urban Ethiopia: A qualitative study
Source: Matern Child Nutr. 2023 May 7;20(Suppl 5):e13527. doi: 10.1111/mcn.13527 (PMC11258761; doi:10.1111/mcn.13527)
Supplement: Supplementary file 1 — Supporting information. [file MCN-20-e13527-s001.docx]

**Table S1** Example of identified issues to be addressed by future interventions

| **Domains** | **Issues that require attention** | **Illustrative quotes** |
| --- | --- | --- |
| **Dietary behaviors of adolescents** | Consumption should be informed by blood type | *“It is healthy to have fruits and vegetables. But, I would suggest people to check their blood type and have their dietary choices based on what is compatible to their blood type…”*  *“My blood type is A…and I know avocado is good for me. People who have a blood type of “O” are not advised to eat Avocado”* |
|  | Avocados are high in fat and can cause overweight if consumed daily | *“…consuming avocado regularly can lead to overweight”* |
|  | There is no such thing as unhealthy diet | *“I do not think that there is such a thing as unhealthy diet; otherwise, we would have not survived…”* |
|  | All soft drinks are unhealthy except *Mirinda* which can prevent anemia | *“Doctors order Mirinda [sugar-sweetened beverage] for sick patient with no appetite … it must be good for patients.”*  *“Mirinda is given after blood donation, it must be good to replenish the blood donated; hence, good if given for the anemic…”* |
|  | Bringing lunch to school is only appropriate for younger children | *“Why do I bring lunch like kindergarten students…?”* |
| **Social factors** | Peer influence | *You had a quote on stigma or peer pressure about bringing/buying lunch* |
|  | Parents’ influence | *There was a quote on what parents tell the adolescents to eat* |
| **Personal and school food environment** | Dichotomy between physical inactivity and unhealthy diet as causes of overweight/obesity  Both physical inactivity and unhealthy eating should be addressed | *“the students can eat what they want… the main thing is that they need to be physically active”* |
|  | Limited source of nutrition information in schools; limited content in the school curriculum, no school/youth clubs focusing on nutrition and healthy eating; widespread misinformation | *“I learnt about nutrition when I was in grade nine…from biology and physical education textbooks... however, in the new curriculum information on nutrition is included starting from grade six…”* |
|  | Wide availability of unhealthy foods in and around the school | *“Merchants look for a strategic place for their business. Like it is common to access drug stores around hospitals, it is also common to access stationary, kiosks and junk food outlets in school surroundings … In front of the shops, you can find a frying machine. It is difficult to escape these foods if you have money.”* |
|  | Processed packaged foods that are not expired and are purchased from cleaner environments like supermarkets are healthy | *“Packed foods are produced to provide more benefit to us. That is why they are processed in factories and made available to us in wider markets. The chemicals added to them could also be beneficial to our health and I think it is useful to consume packed foods and drinks… it is up to us to check their expiration date and ingredients. If they are not expired, they have the required ingredients, and are stored in a safe environment, it wouldn’t be a problem to consume these foods.”* |
|  | Street vendors targeting students for selling unhealthy foods | *“… to increase their [street-vendors] profits, they use cheap, lower quality oil, and use the same oil repeatedly for two to three days. That is why I am saying fried foods are not healthy and shouldn’t be consumed.”* |
|  | Poor hygiene of street vendors and the increased food safety risk associated with the way the food is prepared and stored | *“… to increase their [street-vendors] profits, they use cheap, lower quality oil, and use the same oil repeatedly for two to three days. That is why I am saying fried foods are not healthy and shouldn’t be consumed.”* |
|  | Limited dedicated space for students to sit and eat | *“there is no sitting space and the place is dusty…we prefer foods that can be eaten on the go”* |
|  | Intermittent water supply making washing hands and foods life fruits challenging | *“the water supply is intermittent, forcing us to choose packed foods over fruit, vegetables and hand-eaten foods like injera”* |
|  | Sponsorship of school events by food industries and vendors of unhealthy foods | *“I remember a burger house that sponsored an event in the school… students competed and those ranked in the top three were invited to have whatever they wanted from the burger house…this was also a promotion for the company,”* |
